# Discovery of the final primitive Frank-Kasper phase of clathrate hydrates

**DOI:** 10.1126/sciadv.adp4384

**Published:** 2024-07-24

**Authors:** Sanehiro Muromachi, Satoshi Takeya

**Affiliations:** ^1^ Energy Process Research Institute (EPRI), National Institute of Advanced Industrial and Science Technology (AIST), 16-1 Onogawa, Tsukuba 305-8569, Japan.; ^2^ Graduate School of Engineering, Yokohama National University, 79-5 Tokiwadai, Yokohama 240-8501, Japan.

## Abstract

In weakly bound materials such as water, one of the three primitive Frank-Kasper (FK) phases, the Z phase, is long absent due to the relatively unstable framework. The Z phase in clathrate hydrate, which is known as the HS-I structure, has now been found by precise tuning of the molecular guest structure. In the crystal structure, the never stabilized combination water cage of two 15-hedra and two 14-hedra formed with its original symmetries, providing sufficient gas capacity to the 12-hedral cages. With the discovery of the final FK clathrate hydrate, guest design now enables engineering of weak interactions in any mix of the three, illuminating how to leverage properties of clathrates in the broadest sense.

## Teaser

The missing Frank-Kasper Z phase in clathrate hydrate has now been found, unveiling how to leverage properties of clathrates.

## INTRODUCTION

Puzzles with chemicals echo a natural curiosity through our experiments with rocks, plastic blocks, and virtual objects in video games, revealing a deep-seated inclination to explore and master the microscopic world of atoms and molecules. Many of porous or macroscopic macromolecular structures even including bubble foams (*1*), not only crystalline materials, are known to belong to Frank-Kasper (FK) phases (*2*) which are puzzles of polyhedra fascinating intense study in various fields. Recent examples include the creation of materials from the FK phases, e.g., alloys (*3*), to macroscopic structures, e.g., functional polymers (*4*–*6*) and large scale crystal engineering of DNA-assisted colloids (*7*–*9*).

Water, in its numerous forms, has long captivated scientific inquiry, ranging from the intricate structures of ice to the complex arrangements of clathrate hydrates. More than 20 water structures have been found and proved to be ice, and new ice structures have recently been found, starting with clathrate hydrates (*10*–*12*) which originally contains guest molecules such as inert gases inside the host water lattices. Among these numerous forms of water, clathrate hydrates represent a particularly fascinating subject due to their beautiful molecular architecture made of the simple triatomic molecule. Beyond their physicochemical interests, clathrate hydrates are known to play pivotal roles in areas such as energy resources and environmental science and technology (*13*, *14*). In their structures, water shows self-assembling around guest substances to make FK-type polyhedral framework. Although they are open to exploring structures with many options for guest substances, their fragile framework of hydrogen bonds still makes it difficult to arrange water molecules as intended, which is a simple, small, and highly stable molecule that does not readily bind to other materials.

**Fig. 1.**
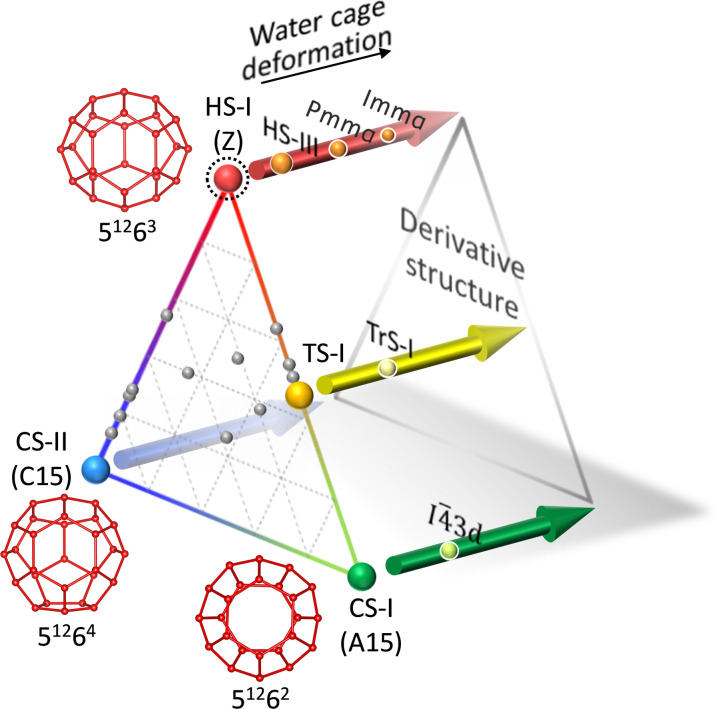
FK-type clathrate hydrates and their derived structures with water cage deformation. Symbols in the brackets show the corresponding FK phase. Arrows show conceptual direction to water cage deformation (*17*). Gray sphere: Other FK phases found in alloys (*15*). The polyhedra show water cages that represent the corresponding FK-based clathrate hydrate: 5^12^6^2^ cage to CS-I; 5^12^6^4^ cage to CS-II; 5^12^6^3^ cage to HS-I. The notation of polyhedra is given in the text.

Among the structures predicted but still awaiting empirical confirmation within clathrate hydrates, the HS-I structure stands out as a notable yet elusive entity. On the FK theory, which is the geometric principle, there are three primitive phases, i.e., A15, C15, and Z, and variety of phases can be obtained as a mixture of these three phases (see Fig. 1) (*15*). The major three structures of clathrate hydrates are the cubic structure-I (CS-I), the cubic structure-II (CS-II), and the hexagonal structure-III (HS-III), which only consist of water (*13*, *16*, *17*). The CS-I and the CS-II correspond to A15 and C15 of the FK phase, respectively (*18*); however, the final primitive FK phase, Z, is still absent in the clathrate hydrates while it is predicted as HS-I (see Fig. 1). The HS-III (*19*) is a truncated form of HS-I of the FK phase. While a total of 27 FK phases have been found in alloys, only 5 have been found in the clathrate hydrates, i.e., CS-I, CS-II, HS-III, the tetragonal-structure-I (TS-I), and its derived structure (TrS-I) (*17*). This means that the art of periodically arranging only tens to hundreds of water has not yet been established. The FK-based thermodynamic theory developed by Matsumoto and Tanaka (FK-MT theory) (*18*, *20*) revealed the reason for the scarce variety of the structures, that is, the free energy of the HS-I framework is relatively high compared to the other two primitive FK clathrate hydrates, i.e., CS-I and CS-II. Similarly, Group 14 element clathrates, such as C, Si, Ge, and Sn clathrates, are under similar host lattice constraints as for clathrate hydrates (*20*). If a full range of the FK triangle can be explored, then the extensions of their unique properties are highly expected, e.g., improving the efficiency of photovoltaics (*21*), developing room temperature superconducting materials consisting of hydrogen bonds (*22*) and durable lithium-ion batteries (*23*), reducing the thermal conductivity of semiconductors (*24*), improving thermoelectric materials (*25*, *26*), and creating carbon materials to be ultrahard (*27*, *28*) and superconductive (*29*).

Clathrate hydrates are composed solely of hydrogen bonds between water molecules, which are relatively weak bonds, and it has been considered difficult to form HS-I structures in a stable manner. In Fig. 1, behind the FK triangle, the derived structures of which water cages are deformed from the FK-based structures by guest substances are shown. Those of the HS-I are the HS-III of true clathrate hydrate and the orthorhombic *Pmma* and *Imma* of semiclathrate hydrates which contains ionic guest substances in addition to water. As a metastable form, a few papers reported the HS-I structure: Yang *et al.* (*30*) reported a metastable HS-I phase of Xe clathrate hydrate at 40 K which was formed after release from extremely high pressure of 40 GPa; however, this work did not depict the structure of the true form of the HS-I. On the other hand, studies of molecular dynamics suggested the formation of transient 5^12^6^3^ cages (*31*) and metastable phases (*32*, *33*), which are identified to be FK-type clathrate structures containing HS-I. However, in compliance with the stability estimation based on the FK-MT theory (*18*), the HS-I structure has not appeared in any stable form to the present. Here, we report the formation of stable HS-I clathrate hydrates by tuning the molecular structure of the ionic guest.

## RESULTS

**Fig. 2.**
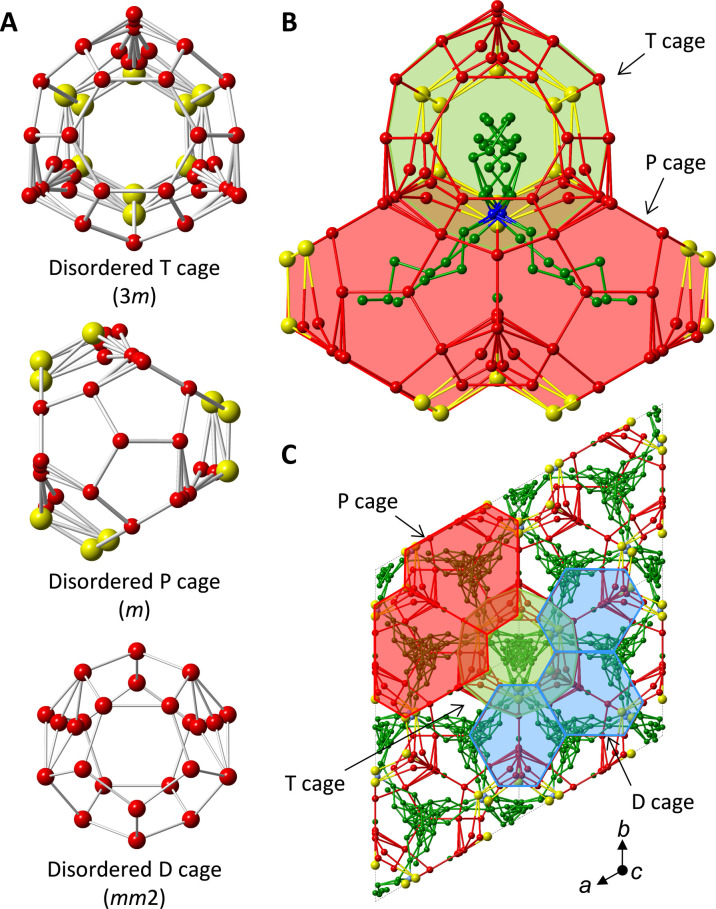
Structural characteristics of the present HS-I structure. Hydrogen atoms are omitted for clarity. Letters in the brackets show point group symmetry of the cage. Polyhedra: Blue, D cage; green, T cage; red, P cage. Sphere: Red, oxygen of water; yellow, chlorine; blue, nitrogen; green, carbon. (**A**) The three types of cages with disorders. (**B**) The T^2^P^2^ cage occupied by the N4446 cation. (**C**) A view of 2 × 2 × 2 unit cell from *c* axis.

**Fig. 3.**
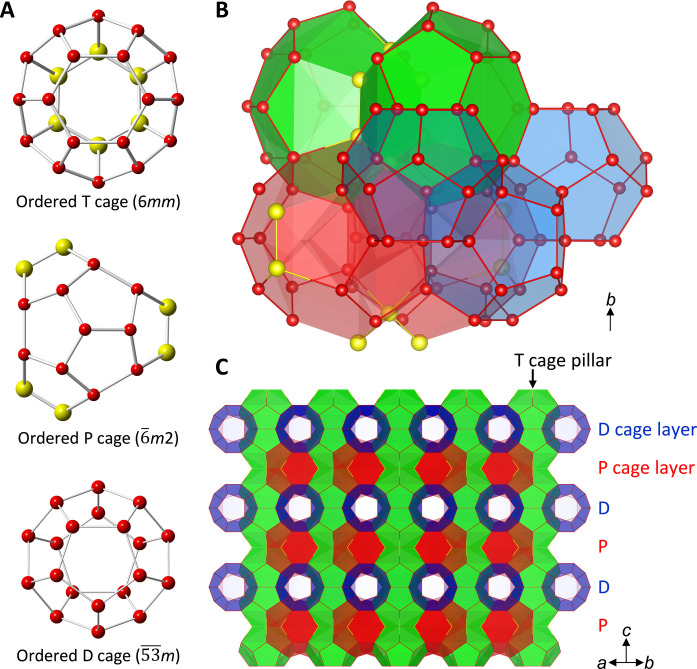
Water cages with ideal order. (**A**) Ideally ordered form of the three types of cages. (**B**) The unit of cage combination. (**C**) Layered structure of the HS-I.

We succeeded in formation of the stable HS-I hydrate with tri-*n*-butyl, *n*-hexylammonium chloride (N4446Cl, where N, 4, and 6 denote the central nitrogen, *n*-butyl chain, and *n*-hexyl chain, respectively) under gas pressures of both methane and carbon dioxide and also atmospheric pressure. The single crystals of the N4446Cl + CH_4_ hydrates are shown in fig. S1. Crystallographic information file (CIF) is available free of charge with Cambridge Crystallographic Data Centre (CCDC) number 2340863 via internet. The present structure was determined to be hexagonal with $P\bar{6}$*m*2 space group. The lattice size was *a* = 12.170 Å and *c* = 12.496 Å, which is close to that estimated by Jeffrey (*16*), *a* = 12.5 and *c* = 12.5 Å. Figure 2 shows a whole structure of the present HS-I hydrate. The prototype of HS-I structure is a hexagonal structure which consists of dodecahedron (D or 5^12^), tetrakaidecahedron (T or 5^12^6^2^), and pentakaidecahedron (P or 5^12^6^3^) where 5 and 6 denote pentagonal and hexagonal faces, respectively (*16*). Its cage composition is 3D + 2T + 2P, where superscript denotes the number of faces in polyhedron. Although the present cages have many disorders, they can be ordered by choosing symmetric positions for their vertices. Figure 3 shows the symmetries of the T and P cages in the ideally ordered forms. They are 6*mm* and $\bar{6}$*m*2, respectively, and identical with the predicted true forms (*16*, *17*). The whole cage combination of the unit cell is 6D·2T^2^P^2^. In pairs of T cages (T^2^), the butyl chains settled like an anchor, allowing the rest of the two arms to be positioned in any two of the six P cages that surround these T cages. The other butyl and hexyl chains occupied the P cages. The nitrogen atom of the cation locates at the junction of T^2^P^2^ cage; however, in the present structure, it can occupy three of six vertices of hexagonal face where two T cages coupled. These vertices are strongly disordered points where nitrogen of the cation and chlorine anion appeared alternately. The long hexyl chain fits into the P cage, and it may stabilize the P cage in a natural form compared to the P cage in the orthorhombic structure. Figure 3C shows a view of the structure from [1, 1, 0] plane. The T cage aligned toward the *c* axis like pillar of the structure. On the *a*-*b* plane, the D cage and the P cage alternately layered.

**Fig. 4.**
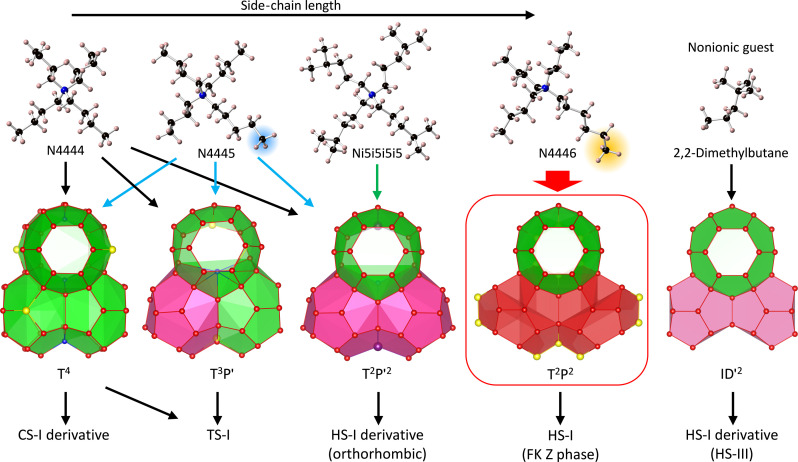
The large cage selectivity in semiclathrate hydrates and inclusion of quaternary alkylammonium cations and a large nonionic guest. Only N4446Cl can stabilize T^2^P^2^ cage to form HS-I structure. T, tetrakaidekahedral cage; P′, pentakaidecahedral cage in the distorted form with the symmetry of *m*; P, pentakaidecahedral cage in the true form with the symmetry of $\bar{6}$*m*2. Sphere color: Red, oxygen of water; yellow, chlorine; purple, bromine; blue, nitrogen of cation; black, carbon; light salmon, hydrogen. T^4^ and T^3^P, tetragonal *P*4_2_/*m* hydrate (*57*); T^2^P^2^, orthorhombic *Pmma* hydrate (*35*, *41*); ID′^2^, HS-III hydrate (*58*) where I and D′ denote 5^12^6^8^ icosahedral cage and 4^3^5^6^6^3^ dodecahedral cage; T^2^P^2^, HS-I in this study.

The disordered P cage has a symmetry of *m*. The symmetry of the ideally ordered P cage was $\bar{6}$*m*2, which was identical with that originally predicted by Jeffrey (*16*) and that found in Br_2_ true clathrate hydrate (*34*). Contrary, the cage symmetry of the P cage found in the conventional orthorhombic *Pmma* (*35*) and *Imma* (*36*) structure was *m*, which is quite lower than the ideally ordered one. In the ordered P cage as shown in Fig. 4C, imaginary Cl─Cl bonds appeared three times at an equivalent position, where simultaneous occupancy of both Cl sites cannot actually happen. However, x-ray crystallography allows observing the HS-I structure with this symmetric P cage using site occupancy by averaging data over time and space. It is noteworthy that the anion may play an important role in forming the ordered P cage by supporting with longer Cl─O bond distances than O─O which cannot be realized only by water molecules. The T cage has also strong disorders in its lattice. In the ordered form, one of its two hexagonal faces only consists of chlorine, and these chlorine ions are identical with that formed ordered P cage, i.e., sites labeled as Cl2 and Cl06 in the provided CIF file (see the Supplementary Materials). Differing from the conventional semiclathrate hydrate structures (*35*, *36*), only one type of D cage is found in the present HS-I structure.

The gas capacity of the present HS-I hydrate was confirmed by gas uptake tests and phase equilibrium measurements. The D cage occupancy (or conversion) data for the present HS-I hydrate obtained from the bulk phases were given in fig. S4 and table S2. In comparison with the CS-II hydrate, the present HS-I hydrate showed better occupancy for CO_2_ gas, while it had the slightly lower occupancy for CH_4_ gas. The orthorhombic *Pmma* hydrate of tetra-*n*-butylammonium bromide (TBAB), which is a widely used ionic guest, clearly showed the lowest gas capacity likely due to its low symmetric structure. The three phase (gas-hydrate-liquid) equilibrium data also supported the higher gas capacity of N4446Cl hydrates than those of TBAB (see the Supplementary Materials). Another important finding is that the equilibrium temperatures for the N4446Cl + CO_2_ hydrates are higher than those with CH_4_. Since the molecular size of CO_2_ is slightly large for the D cage, the conventional promoted clathrate hydrates showed irregular capture properties of CO_2_ such as the inconsistent cage occupancies in CS-II hydrates (*37*, *38*) and the scarce HS-III hydrate formation under CO_2_ pressure (*39*, *40*). Therefore, it can be noted that the potential gas capacity of the HS-I hydrate was almost fully activated with both CH_4_ and CO_2_ gas.

The preservability of the present HS-I hydrates was evaluated by powder x-ray diffraction (PXRD) measurements. The temperature profiles of PXRD pattern (see fig. S3) for N4446Cl + CH_4_ or CO_2_ hydrate under vacuum conditions did not change from 93 to 268 K for CO_2_ and to 258 K for CH_4_, which indicated that the N4446Cl + gas hydrates can be preserved over 258 K. This suggests that the N4446Cl hydrates can keep holding gases up to near the freezing point of ice which is widely available in the existing cold chain; therefore, this material is readily used for the purposes of gas storage and transportation.

## DISCUSSION

### Water material science

The relationship between molecular structures of quaternary ammonium cations and the combined cages which can be stabilized by the cations is shown in Fig. 4, e.g., well symmetric ions such as TBA (or N4444) (*35*) and tetra-*i*-pentylammonium (Ni5i5i5i5) (*16*) and partly asymmetric tri-*n*-butyl, *n*-pentylammonium (N4445) (*41*) cations, where, in abbreviation, N, 4, 5, and i5 denote nitrogen, *n*-butyl, *n*-pentyl, and *i*-pentyl, respectively. The *n*-hexyl chain is the only guest moiety that can stabilize the true form of the P cage with the $\bar{6}$*m*2 symmetry required for the HS-I structure, which has never been stabilized by the other guests. The distortions in the hydrogen bonds of the water molecules in these structures are caused by incorporation of the cations, especially in the hexagonal faces of the P cage in the true form to be the distorted form (P′ cage). The present results found that *n*-butyl and *i*-pentyl chains are not large enough to stabilize the P cage which has an ideally symmetric shape. In other words, such minute size difference in hydrophobic part, e.g., *n*-hexyl and *n*-butyl, may determine the whole structure of the clathrate hydrates. In Fig. 4, the ID′^2^ cage of the HS-III structure, which is one of the three major structures of clathrate hydrates, was together shown, where D′ cage is 4^3^5^6^6^3^, the 4 denotes square face. The I cage is the combined T^2^ cage, which also absorbs a part of the P cage space which were sandwiched between the T^2^ cage from the perspective of the direction perpendicular to the hexagonal face of the T cage. As a result, the D′ cage takes the shape of the part of the original P cage that was shaved off by the I cage. When the I cage can be stabilized by occupancy of a large molecule such as 2,2-dimethylbutane, the HS-III structure forms, but to support HS-III structure, guest gas occupancy in the D and the D′ cages is necessary. Since CO_2_ inclusion in the HS-III structure has been unclear even under support by CH_4_ (*39*), CO_2_ seems to be an unpreferable guest for the D′ cage which is required to support this HS-I–derived structure. In the present HS-I structure, since the truly formed P cage was stabilized by the cation, CO_2_ is not required to occupy the D′ cage but only the D cage.

As Jeffrey monologued that “the tetra-*n*-butyl and iso-amyl ammonium cations appear to provide the ‘best’ and in fact the ‘only fit’ consistent with formation” (*16*), no cation has yet appeared that gives a higher melting temperature at ambient pressure. However, the present discovery provides a new aspect, that is, fitness of cation into the orderly hydrogen bond network does not appear to melting temperature of hydrates but gas capacity. What we have seen so far in semiclathrate hydrates may have been specialized for cation inclusion. The clathrate hydrates are known as nonstoichiometric compounds, which indicate that some part of the cages can be left empty (*13*, *17*). The excessive fitting of the cation to the derived structures may, however, greatly reduce the gas capacity, as the framework no longer requires support by gas. Conversely, a melting point of 300 K had already been successfully achieved in exchange for a half of the potential gas capacity. From a similar perspective, the HS-III structure can be regarded as a derived structure which sacrificed T cage and P cage to obtain gas capacity of small gases such as methane. These substantial changes in properties are not limited to the frame of the FK phase as the present HS-I and the derived structures (*Pmma* and *Imma*) both belong to the HS-I in the FK theory.

Another important finding is that this new structure achieves the first inversion of gas capture preference in the D cage for CO_2_ and CH_4_. This breakthrough challenges the previously accepted concept that clathrate hydrate selectivity cannot be drastically altered for similarly sized gas molecules, paving the way for innovative applications in gas separation technologies. In the future, revisiting this HS-I structure may enable attempts to customize it in these specific directions. As the final piece for the FK clathrate hydrate was obtained by the present study, by designing guest shape, it may be possible to form any mixture of primitive three FK phases to engineering the weak interactions and to reinforce the properties of clathrate hydrate for practical applications such as heat storage, CO_2_ capture and storage, and gas transportation.

### FK phase in weakly bound materials

Over 60 years ago, Frank and Kasper (*2*) indicated geometric constraints on the FK phases, and Matsumoto and Tanaka (*18*, *20*) formed a hill of thermodynamic potential on the FK triangle map to find that the FK phases are strongly constrained by the host constituents and cannot be stabilized by simple geometric consistency alone. Probably because of this reason, Z phase in self-assembling soft matter had been missing, while it was found a few years ago by accurately adjusting length and interaction between components (*42*). This study showed that connecting some key blocks of potentially unstable phase by precursors, the T^2^P^2^ cage by N4446Cl in this case, can realize it in a stable form. The present technique sheds light on how the clathrates in the broadest sense can be exploited to extend their unique properties. Fortunately, Group 14 elements have options for the host elements and dopants that can cooperate to overcome geometric constraints, similar to how the host structure of the HS-I clathrate hydrates is achieved with the help of ion.

In a category of the three primitive FK phase in clathrate hydrates, the present discovery is the first in about 70 years, since the CS-II structure had been confirmed by x-ray diffraction in 1951 (*43*). After a series of explorations in conditions close to our living environment, the search for new structures in water has been to keep the structure of water under extreme conditions, such as ultrahigh pressure on the order of gigapascal or high vacuum, and cryogenic temperatures. An unexpected outcome was that such primitive structure could be formed under conditions close to ambient temperature and pressure. As we have achieved this with ordinary laboratory setup such as flasks and test tubes in the lab, the future opens the horizon for ecological research and application development of materials that are within our reach.

## MATERIALS AND METHODS

### Materials and synthesis

Water (deionized, sterilized by an ultraviolet lamp, and filtrated by hollow fiber and activated carbon), methane (>99.995% purity, Tokyo Gas Chemical Co. Ltd.), and carbon dioxide (>99.995%, Taiyo Nippon Sanso, Co.) were used for hydrate formation. N4446 cation was synthesized from a reaction of tri-*n*-butylamine and 1-bromohexane in nitrobenzene through the method that follows the previous study (*41*). N4446Cl was obtained by ion exchange with resin (IRA402BL-Cl, DuPont de Nemours Inc.). Yield: 30%. The product was a viscous yellow liquid at room temperature. Product contains 4 mass% of impurities of byproducts of tri-*n*-butylamine. Yield: ^1^H nuclear magnetic resonance (500 MHz, chloroform-D) δ 3.20 to 3.30 (m, 8H,–N–*CH*_2_–), 1.54 to 1.64 (m, 8H,–N–CH_2_–*CH*_2_–), 1.19 to 1.43 (m, 12H,–N–CH_2_–CH_2_–*CH*_2_–,–N–CH_2_–CH_2_–CH_2_–*CH*_2_–CH_2_–CH_3_,–N–CH_2_–CH_2_–CH_2_–CH_2_–*CH*_2_–CH_3_), 0.86 to 0.96 (m, 9H,–N–CH_2_–CH_2_–CH_2_–*CH*_3_), and 0.77 to 0.84 (m, 3H,–N–CH_2_–CH_2_–CH_2_–CH_2_–CH_2_–*CH*_3_).

### Single-crystal x-ray diffraction

Crystal samples of the N4446Cl + CH_4_ hydrate were formed in a high-pressure cell which had a volume of ~100 cm^3^ and optical window to observe inside. About 3 g of an aqueous solution of ionic guest was injected into the cell. The cell was pressurized with methane gas at a desired pressure. After a few days for crystal growth of hydrate, the crystals were taken out from the cell. The picture of the crystal was provided in fig. S1. The formation conditions for the N4446Cl + CH_4_ hydrate was 3 MPa and 280 K. Detail procedures are found elsewhere (*36*).

Diffraction measurements were performed with a device (XtaLabSynergy-S, Rigaku Co. Ltd.). The x-ray source was Cu *K*α. The crystal size was 0.01 mm × 0.01 mm × 0.01 mm. The structure was solved and refined by Shelx program (*44*) with assistance of Olex software (*45*). The structure model was visualized with VESTA (*46*) and Crystal Maker software. The symmetries of hydrate cages were determined by Jmol software (*47*). The final indices for the present structure were *R*_1_ = 6.5% and w*R*_2_ = 18.6%. The chemical formula obtained from the single crystal x-ray diffraction (SCXRD) data were 0.9 N4446Cl·37.6 H_2_O·0.5 CH_4_. CIF is available free of charge with CCDC number 2340863 via internet. Given the high symmetry of the HS-I, most of the crystal samples were appeared as twinned crystal or polycrystal with a large pseudo-tetragonal lattice, e.g., 12 Å × 12 Å × 100 Å. The reduction data with this large lattice led wrong structure models with convergences to high *R* value, e.g., *R*_1_ > 20%.

### Phase equilibrium measurement

The phase equilibrium measurements for the present N4446Cl + (CH_4_ or CO_2_) hydrate were performed in the similar setup with the gas uptake tests. Once the reactor was charged with the gas up to a prescribed pressure, the cell was cooled down to form hydrate. Because of the hydrate formation, the pressure decreased. Subsequently, the water bath temperature was stepwise increased to dissociate hydrate. At each temperature step, the pressure was equilibrated by stirring, which usually took 2 to 8 hours. The used temperature step was 0.1 K. The aqueous concentration of the N4446Cl solution was measured by a Karl Fischer titrator. The detail setup and procedures were described elsewhere (*48*).

The equilibrium conditions for these hydrates obtained in this study were provided in fig. S2 and table S1. While the melting temperatures of semiclathrate hydrates of N4446Br and N4446Cl were below 275 K, under gas pressure of methane or carbon dioxide, the equilibrium temperature drastically increased. The pressure-temperature (*P*-*T*) slope of the equilibrium curve represents gas capacity of the hydrate phase based on the Clausius-Clapeyron equation (*13*). In fig. S2B, both of the N4446Br and N4446Cl indicates that their *P*-*T* slopes are smaller than those of TBAB (*49*, *50*) and are steeper than tetrahydrofuran (THF) (*51*) and no additive systems (*52*, *53*).

### Preservability test by PXRD

PXRD was performed with a device (40 kV, 40 mA, Ultima III, Rigaku, Japan) in a cryo-chamber. The N4446Cl + CH_4_ hydrate samples were ground into a fine powder at a temperature below 100 K under N_2_. The powder sample was loaded into a copper specimen holder with a depth of 0.50 mm and then placed in the cryo-chamber attached to the x-ray diffractometer. PXRD measurements were performed in the θ/2θ step scan mode with a step width of 0.02° by using Cu *K*α radiation, where θ denotes diffraction angle. PXRD measurements were performed under a vacuum condition for 110 min, with the temperature increasing every 15 from 93 K.

The temperature profiles of PXRD pattern for N4446Cl + CH_4_ or CO_2_ hydrate are shown in fig. S3. The PXRD pattern agrees well with the hexagonal structure with *P*$\bar{6}$*m*2 space group which was determined by the SCXRD. The profiles obtained under vacuum conditions did not change from 93 to 268 K for CO_2_ and to 258 K for CH_4_, which indicated that the N4446Cl + gas hydrates can be preserved over 258 K. This suggests that the N4446Cl hydrates can keep holding gases up to near the freezing point of ice without highly technical processes such as pelletizing and high dehydration. Since such preservation temperature is widely available in the existing cold chain, this material is readily used for the purposes of gas storage and transportation.

### Gas uptake test

Gas uptake tests were performed with a high-pressure cell which was equipped with a mechanical stirrer to convert the most part of solution to hydrate. The cell inner volume including tubing was determined to be 113.0 cm^3^. About 30 g of aqueous solutions was subjected to the tests. Gas uptake by the hydrate was calculated by density differences between before and after hydrate formation. Density of the gas was calculated with the experimental pressure and temperature conditions by REFPROP 10 (*54*). The volumes of the subjected aqueous solutions were altered by mass of solution in gram unit. The detail procedures are available elsewhere (*55*). The D cage occupancy (or conversion) can be calculated by the following equation where the aqueous solutions charged in the reactor were assumed to be fully converted to the hydrates.

$$\theta =\left(\frac{n_{w}^{H}}{n_{g}^{H}}\right)/\left(\frac{N_{w}^{\text{UC}}}{N_{D}^{\text{UC}}}\right)$$

where θ, *N*, *n*, H, UC, g, D, and w denote cage occupancy, number of molecules or cages, moles, hydrate phase, unit cell, gas, D cage, and water. Note that the presently considered structures, i.e., the CS-II, the orthorhombic *Pmma* and *Imma*, and the HS-I structures, D cages are only available for gas occupancy. $N_{w}^{\text{UC}}$ and $N_{D}^{\text{UC}}$ for these structures with the ideal order are (136, 16), (76, 6), and (76, 6), respectively.

The obtained D cage occupancy data for the present HS-I structure hydrates were given in fig. S4. In this figure, the results for TBAB and THF hydrates were together shown for comparison. For CH_4_ gas, the present HS-I hydrate showed the 69% of the D cage occupancy at 10 MPa, and the hydration number approached to the stoichiometric number, i.e., 38 (water)/3 (D cage) = 12.7. For the CS-II hydrate of THF, the D cage occupancy was 86% at 10 MPa, which means that this CS-II hydrate had higher gas capacity than HS-I as expected from its stoichiometry, i.e., 136 (water)/16 (D cage) = 8.5. The gas capacity of the orthorhombic *Pmma* hydrate of TBAB was 42%, which was about half of the HS-I and the CS-II hydrates. For CO_2_ gas, the present HS-I hydrates showed the highest D cage occupancies, i.e., 32 to 49%. This is slightly lower than that in the CS-I hydrate which was reported to be 0.7 (*56*). The *Imma* hydrate of TBAB showed the 20 to 28% of occupancy, which were about half of the N4446Cl hydrates. The CS-II hydrate of THF indicated peculiar behavior: Occupancy decreases with pressure, as well as the inconsistently reported data, i.e., 0.3 to 0.6 (*37*, *38*).

## Supplementary Materials

### This PDF file includes:

Figs. S1 to S4 Tables S1 and S2 Nomenclature
