# Supplementary material for: Discovery of the final primitive Frank-Kasper phase of clathrate hydrates
Source: Sci Adv. 2024 Jul 24;10(30):eadp4384. doi: 10.1126/sciadv.adp4384 (PMC11268421; doi:10.1126/sciadv.adp4384)
Supplement: Supplementary file 1 — Figs. S1 to S4 Tables S1 and S2 Nomenclature [file sciadv.adp4384_sm.pdf]

Science Advances
AAAS

Supplementary Materials for

Discovery of the final primitive Frank-Kasper phase of clathrate hydrates

Sanehiro Muromachi and Satoshi Takeya

Corresponding author: Sanehiro Muromachi, muromachi-sanehiro-sf@ynu.ac.jp

Sci. Adv. **10**, eadp4384 (2024)
DOI: 10.1126/sciadv.adp4384

This PDF file includes:

Figs. S1 to S4
Tables S1 and S2
Nomenclature

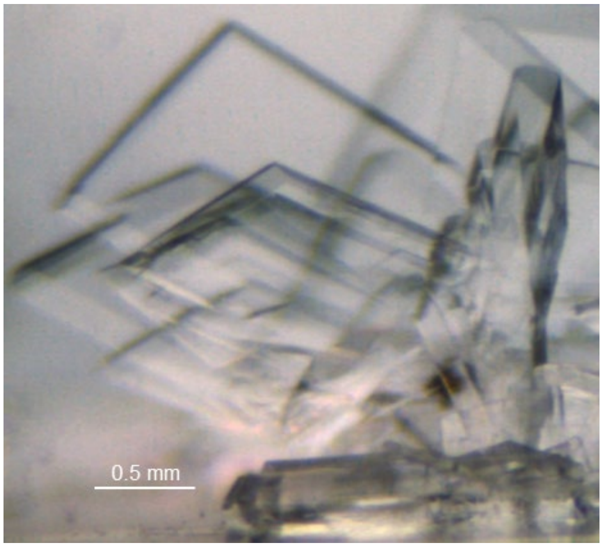

Fig. S1.
The single crystals of the N4446Cl + CH_4 hydrate formed at 3 MPa and 280 K.

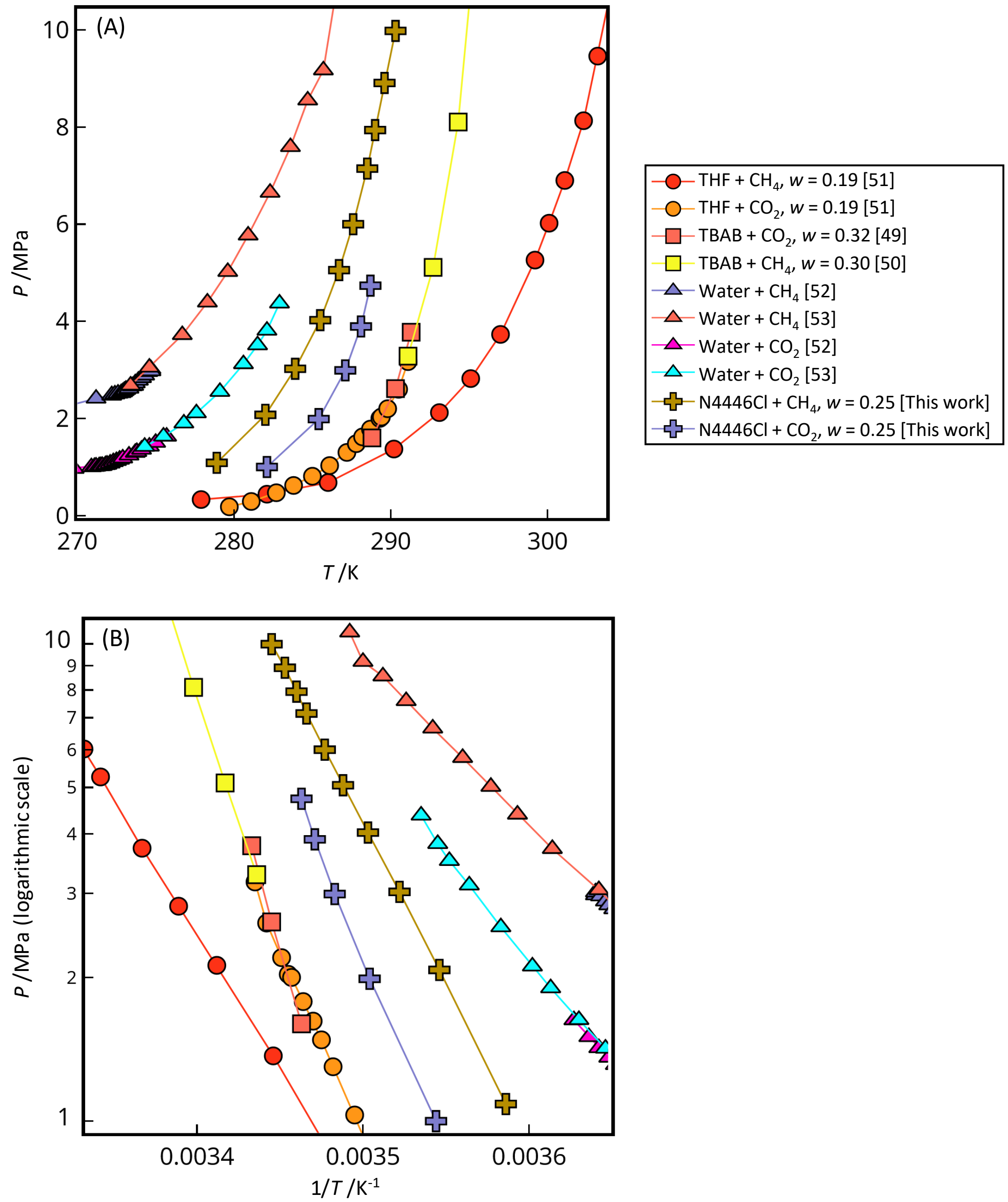

Fig. S2.

Equilibrium pressure and temperature conditions for N4446Cl hydrates under gas pressure of CO_2 and CH_4. (**A**) *P*–*T* data in linear scale. (**B**) *P*–1/*T* data in logarithmic scale.

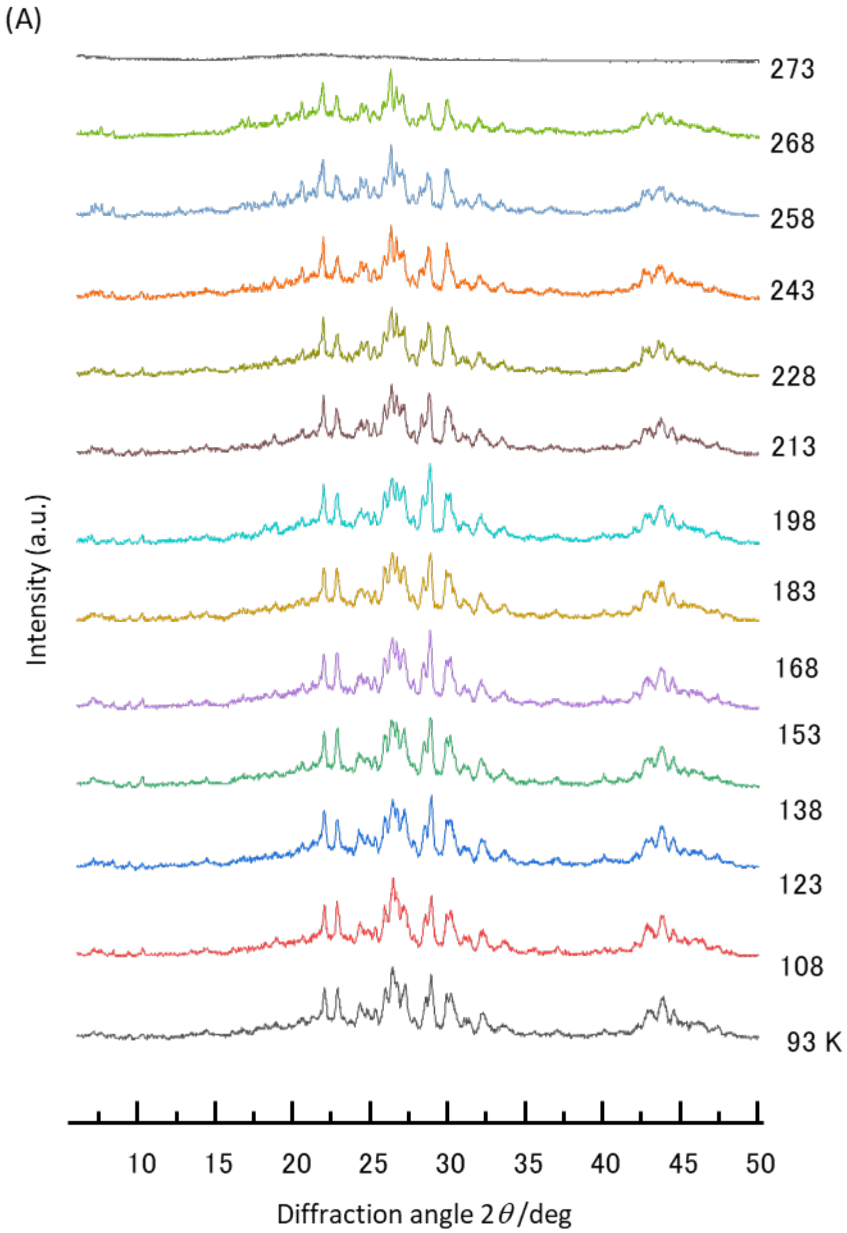

Fig. S3.

Preservability data for the N4446Cl hydrates. (**A**) N4446Cl + CO_2 hydrate. (**B**) N4446Cl + CH_4 hydrate.

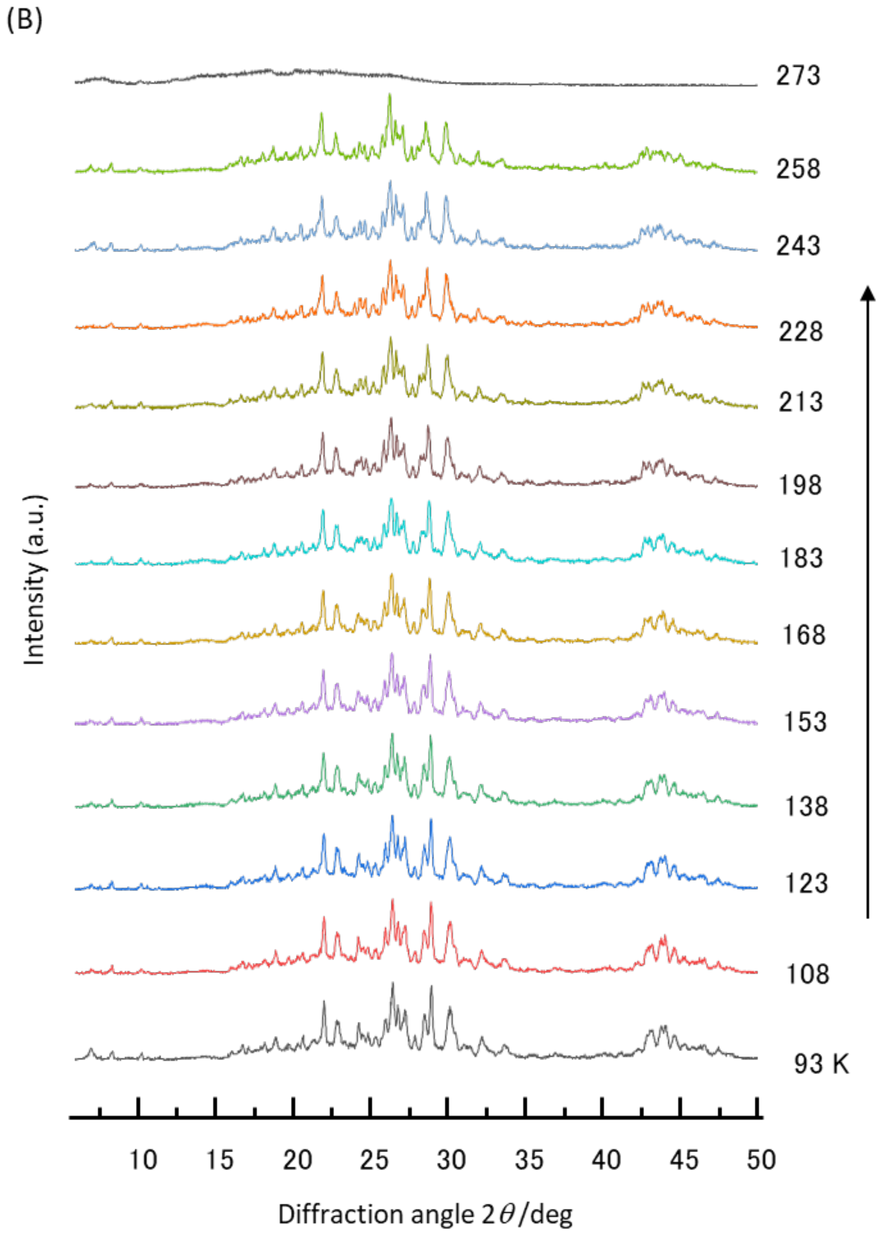

Fig. S3.

(*continued*) Preservability data for the N4446Cl hydrates. (**A**) N4446Cl + CO_2 hydrate. (**B**) N4446Cl + CH_4 hydrate.

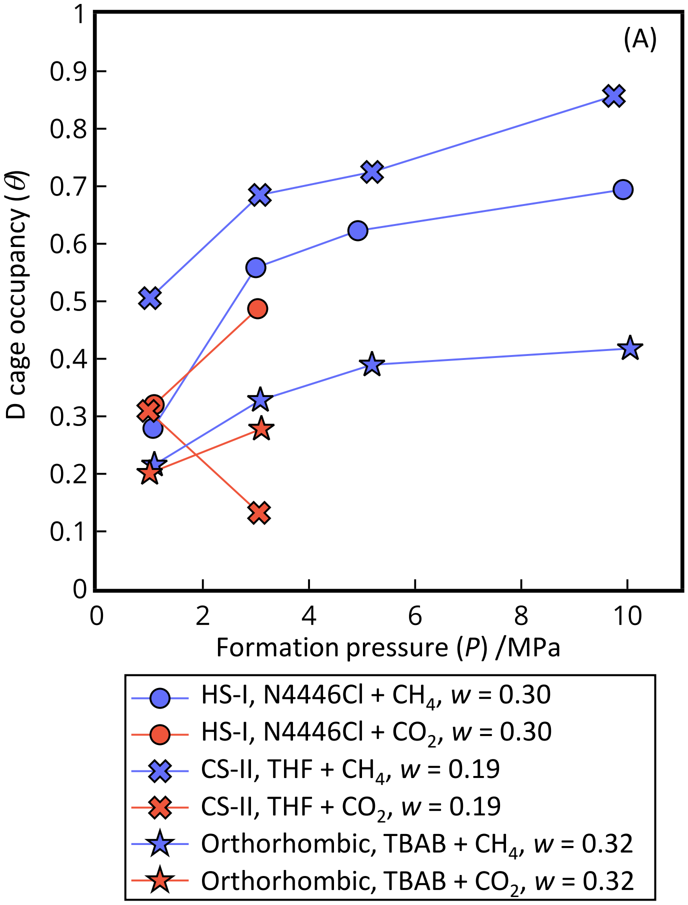

Fig. S4.

Results for the gas uptake tests. (**A**) D cage occupancy. (**B**) Hydration number for guest gas.

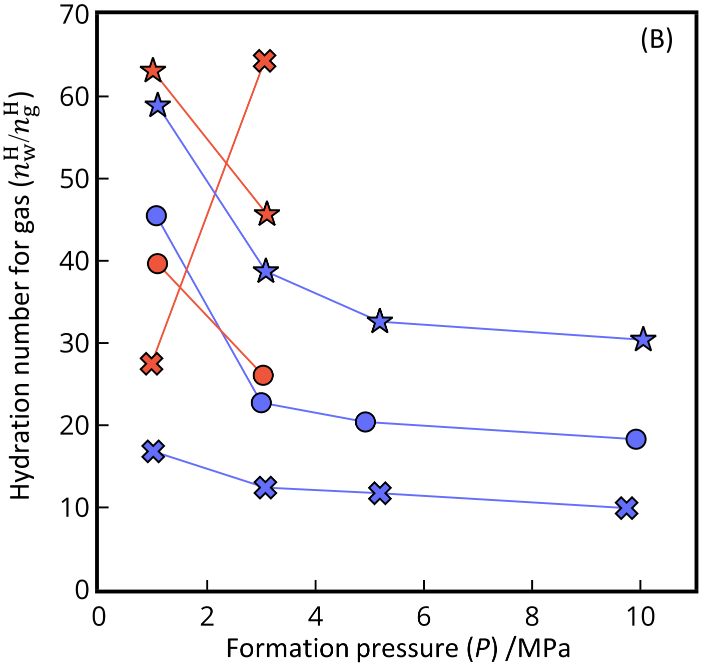

Fig. S4.

(*Continued*) Results for the gas uptake tests. (**A**) D cage occupancy. (**B**) Hydration number for guest gas.

Table S1.

Phase equilibrium data for the N4446Cl + (CH_4 or CO_2) hydrate.

Gas	*w*	*U*(*w*)	*x*	*U*(*x*)	*T*	*P*	*U*(*T*)	*U*(*P*)	
								Lower	Upper
					/K	/MPa	/K	/MPa	/MPa
CH_4	0.250	0.02	0.0193	0.002	278.9	1.09	0.1	0.01	0.01
					282.0	2.07	0.1	0.01	0.01
					283.9	3.02	0.1	0.01	0.02
					285.5	4.02	0.1	0.01	0.03
					286.7	5.05	0.1	0.01	0.02
					287.6	6.00	0.1	0.01	0.02
					288.5	7.14	0.1	0.01	0.05
					289.0	7.94	0.1	0.01	0.11
					289.6	8.91	0.2	0.01	0.00
					290.3	9.98	0.2	0.01	0.09
CO_2	0.250	0.02	0.0193	0.002	282.1	1.00	0.1	0.01	0.02
					285.4	1.99	0.1	0.01	0.01
					287.1	2.99	0.1	0.01	0.02
					288.1	3.89	0.1	0.01	0.03
					288.7	4.73	0.1	0.01	0.05

Table S2.

Table S2. The present data for gas uptake tests.

Gas	Salt	Hydrate		Aqueous solution				Formation conditions			Hydrate phase		
		Structure	N_w^{UC}/N_D^{UC}	w	x	n_w^{Aq}/n_p^{Aq}	m_{aq}	P	T	$\triangle T_{sub}$	n_g	n_w^H/n_g^H	θ
				(mass fraction)	(mole fraction)		g	MPa	K	K	mmol		
CH_4	N4446Cl	HS-I	12.7	0.300	0.0246	39.6	29.58	1.060	272.3	6.9	25.3	45.5	0.28
								2.998	279.1	5.0	50.6	22.7	0.56
								4.921	281.0	5.2	56.4	20.4	0.62
								9.920	284.8	5.3	62.8	18.3	0.69
	TBAB	Orthorhombic *Pmma*	12.7	0.320	0.0256	38.0	29.73	1.086	282.9	5.2	19.1	58.9	0.22
								3.083	286.7	4.5	29.0	38.7	0.33
								5.187	285.6	6.6	34.4	32.6	0.39
								10.053	289.7	5.5	36.9	30.4	0.42
	THF	CS-II	8.5	0.191	0.0557	17.0	30.51	1.002	282.7	4.4	81.5	16.8	0.51
								3.074	290.7	4.4	110.4	12.4	0.68
								5.189	292.7	5.4	116.8	11.7	0.72
								9.745	298.7	6.4	138.1	9.9	0.86
CO_2	N4446Cl	HS-I	12.7	0.300	0.0246	39.6	29.58	1.082	276.4	5.7	29.0	39.7	0.32
								3.033	283.4	3.7	44.1	26.1	0.49
	TBAB	Orthorhombic *Imma*	12.7	0.320	0.0256	38.0	29.73	0.997	283.7	4.5	17.8	63.1	0.20
								3.103	286.8	4.4	24.6	45.7	0.28
	THF	CS-II	8.5	0.191	0.0557	17.0	29.90	0.971	281.8	4.4	48.9	27.5	0.31
								3.059	284.8	5.4	20.9	64.3	0.13

Nomenclature

w	Suffix for water
p	Suffix for promoter (N4446Cl, TBAB and THF)
g	Suffix for gas (CH_4 and CO_2)
H	Hydrate phase
Aq	Aqueous phase
D	Suffix for D cage
UC	Unit cell
P	Pressure
T	Temperature
$\triangle T_{sub}$	Subcooling temperature
w	Concentration of promoter in aqueous phase in mass fraction
x	Concentration of promoter in aqueous phase in mole fraction
n	Molar amounts
m	Mass
N	Moles
θ	D cage occupancy or diffraction angle for PXRD
U	Extended uncertainty with 95% reliability
